# Gold nanoparticle ring arrays from core–satellite nanostructures made to order by hydrogen bond interactions†

**DOI:** 10.1039/d2na00204c

**Published:** 2022-04-23

**Authors:** Yingying Cai, Wentao Peng, Philipp Vana

**Affiliations:** Institut für Physikalische Chemie, Georg-August-Universität Göttingen Tammannstrasse 6 37077 Göttingen Germany pvana@uni-goettingen.de

## Abstract

Polyethylene glycol-grafted gold nanoparticles are attached to silica nanoparticle cores *via* hydrogen bonding in a controlled fashion, forming well-defined core–satellite structures in colloidal solution. For separating these complex structures effectively from the parental nanoparticles, a straightforward and easy protocol using glass beads has been developed. The attached gold nanoparticles show unique surface mobility on the silica core surface, which allows for nanoparticle rearrangement into a 2D ring pattern surrounding the silica nanoparticle template when the core–satellite structures are cast to a planar surface. When etching away the silica core under conditions in which the polymer shell fixes the satellites to the substrate, highly ordered ring-shaped patterns of gold nanoparticles are formed. By variation of the size of the parental particles – 13 to 28 nm for gold nanoparticles and 39 to 62 nm for silica nanoparticles – a great library of different ring-structures regarding size and particle number is accessible with relative ease. The proposed protocol is low-cost and can easily be scaled up. It moreover demonstrates the power of hydrogen bonds in polymers as a dynamic anchoring tool for creating nanoclusters with rearrangement ability. We believe that this concept constitutes a powerful strategy for the development of new and innovative nanostructures.

## Abstract 2

Polymer-grafted gold nanoparticles are attached to silica nanoparticles forming core–satellite structures, which rearrange into ring arrays when cast to surface. By etching away the silica core, ring-shaped patterns of gold nanoparticles are formed.

## Graphical abstract

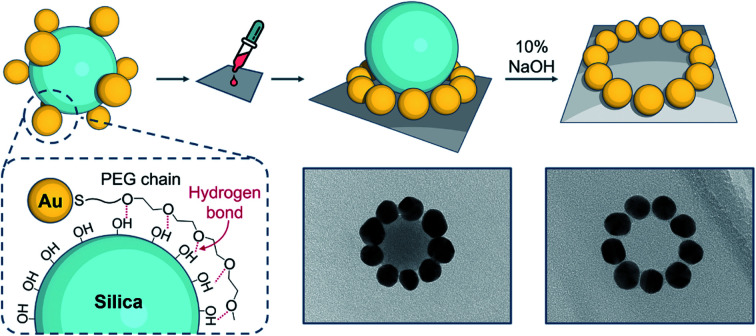

## Introduction

The precise spatial arrangement of plasmonic (gold or silver) nanoparticles (NPs) is of vast importance for manipulating their interaction with light. When the plasmonic NPs are arranged in a closed nanometer distance, their plasmonic resonance coupling will split their plasmonic energy level into different modes based on their interparticle distance and geometry.^1,2^ The optical properties of such “plasmonic molecules” originate from the whole nanostructure and can be predicted by using models that are analogous to molecular hybridization.^2–5^ This paradigm is a powerful tool to guide the spatial structural design of plasmonic nanoassemblies with the aim of creating new ensemble properties,^1,2,6,7^*e.g.*, spectral shift,^8^ Fano resonances,^9,10^ optical magnetism,^6,11,12^ and chirality.^13–16^

In the realm of plasmonic molecules, ring-shaped structures as “aromatic” analogs have been intensively studied for their unique optical properties.^11,12,17–19^ The dipole excitation of the plasmonic nanoparticles in such rings can synergistically interact and form a circular current over the whole nanoassembly (collective modes resembling a coil) inducing a magnetic dipole (so-called magnetic surface plasmon).^12,19^ This unique phenomenon allows the close-packed plasmonic ring-pattern to interact with the magnetic field of the light even in the visible and near-infrared range. This optical magnetism has shown great potential for applications including nanooptics^20–23^ and sensing.^24,25^

Intuitively, creating a circular plasmonic pattern requires a 2D patterning tool or template. The vast majority of examples of plasmonic oligomers are fabricated by top-down electron beam lithography.^17,18^ This method offers great flexibility to create plasmonic structures (*e.g.*, patterned gold disks) with targeted optical properties. However, its spatial resolution is restricted to ∼10 nm,^7^ due to the spot size of the electron beam, thus limiting the applicability of the resulting plasmonic pattern especially for near-field coupling. To increase structural resolution, the bottom-up synthesis^26^ offers a greater variety of the size and geometry of the single plasmonic nanoparticle down to few nanometers. However, the development of colloidal patterning tools for such precise 2D nanotemplates generally requires sophisticated high-end techniques. Wang *et al.* have demonstrated the fabrication of optical magnetic rings (hexamer of AgNPs) assisted by a 2D DNA-origami template.^19^ However, despite the structure perfection, the high costs and low yields of the DNA origami method restrict any scaled-up production of nanostructures. In contrast, polymer-mediated self-assembly of NPs offers highly flexible design options in a scalable manner. Demonstrating this general pathway, functional polymer linkers are *e.g.* used to interconnect nanocomponents in so-called “core–satellite” nanostructure, which can form a 2D pattern of satellite NPs surrounding cores after cast on the substrate.^27–32^ This approach allows for a good control of the core–satellite interparticle distance, whereas the distance between satellites is poorly controlled (often too large) for introducing any plasmonic interaction.

**Fig. 1:**
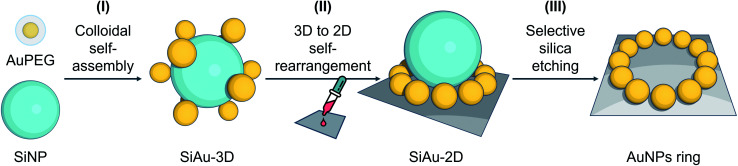
Schematic illustration of the preparation strategy of ring nanopatterns of AuNPs. (I) Colloidal self-assembly of 3D SiAu nanoclusters using hydrogen bond interaction between the PEG shell of AuNPs and the silica surface. (II) 3D to 2D self-rearrangement of AuPEG around SiNP upon the substrate. (III) Selective silica etching without affecting the gold ring nanopattern.

**Fig. 2:**
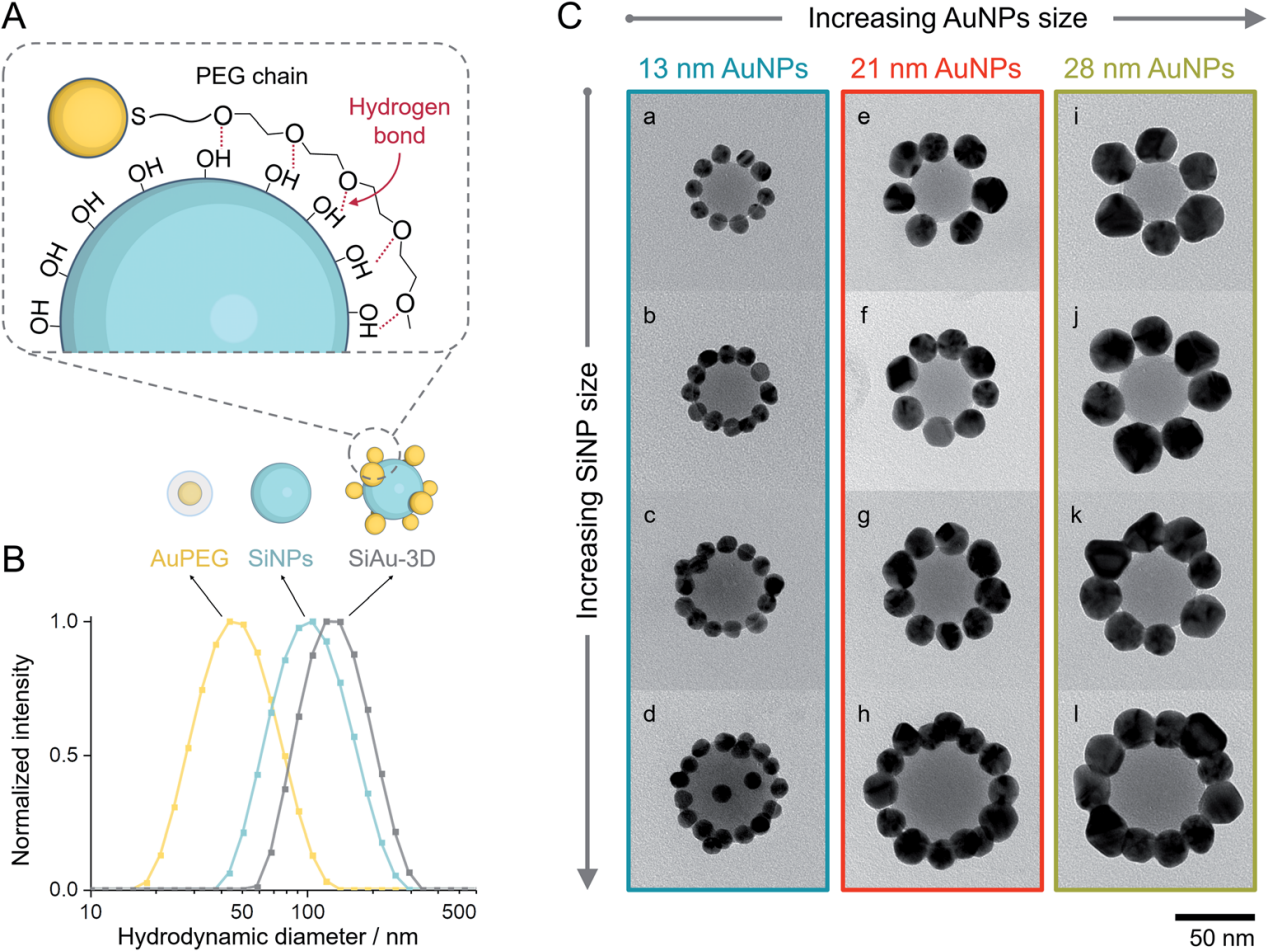
(A) Schematic illustration of the multidentate hydrogen bonding between AuPEG and SiNPs. (B) Hydrodynamic diameters of AuPEG (with 13 nm AuNPs, yellow), SiNPs (46 nm, cyan), and purified SiAu-3D (originated from the same types of AuPEG and SiNPs, gray) measured by DLS. (C) TEM images of SiAu-2D nanoclusters constructed by different AuNPs (13, 21, and 28 nm for each column) and SiNPs (39, 46, 50, and 62 nm for each row). The lower-case letters on the TEM images are corresponsive to the sample labeling.

Based on these considerations, we reconceptualized the framework of the “core–satellite” formation in order to create a highly precise ring pattern of AuNPs. For the first time, hydrogen bond interactions are used to assemble “core–satellite”-like nanoclusters with mobile anchoring sites between nanosatellites and -cores. To do this, we use pristine silica nanoparticles (SiNPs) as core and polyethylene glycol (PEG)-capped AuNPs (AuPEG) as the satellites. As illustrated in Fig. 1, our fabrication strategy comprises three stages: (I) colloidal self-assembly of silica–gold (SiAu) nanoclusters, (II) ring-shaped rearrangement of the AuNPs when the nanoclusters get in contact with a planar solid substrate, and (III) selective etching of the silica cores. The whole nanoengineering process is realized by several innovative functions of the PEG shell upon the AuNPs at different fabrication stages. In the colloidal self-assembly step, the PEG shell serves as a multidentate linker offering a strong adsorption between AuNPs (satellites) and SiNP (core) (Fig. 2A). Once the structure is cast on a planar substrate, the PEG chains work as actuator offering sufficient mobility for AuNPs to rearrange themselves around the spherical SiNP template forming a 2D circular pattern at the meniscus region between the silica core and the substrate. The 2D arrangements were diligently studied in this work in order to develop control tactics for structure prediction. During the final silica template etching, the PEG shell immobilizes the perfect Au ring pattern from any disordering, because of its LCST (lower critical solution temperature) solution behavior (PEG is not soluble in water at high NaOH concentrations). It is worth mentioning that only basic chemicals, lab equipment, and hands-on skills are required to achieve the reported results.

## Results and discussion

### Colloidal self-assembly

As mentioned before, PEG undergoes unspecific adsorption on the surface of silica *via* hydrogen bonds.^33,34^ Exploiting this interaction, we established a method to attach AuPEG onto the surface of spherical SiNPs through a colloidal self-assembly process (Fig. 2A). For brevity, the colloidal silica–AuPEG nanostructure is called SiAu-3D. The fabrication of AuPEG is simply accomplished by mixing commercially available thiol terminated methoxy PEG (6000 g mol^−1^) with an aqueous colloid of AuNPs. In this way, dense polymer brushes of PEG are formed upon the surface of gold *via* strong Au–S covalent bonds.^35^ Both SiNPs and AuPEG colloids can be used as stock solutions with known concentrations for convenient experimental screening.

The colloidal self-assembly of SiNPs and AuPEG into SiAu-3D was conducted in THF at room temperature by simply mixing under sonication and incubation. In this step, AuPEG is added in excess to saturate the SiNP surface with PEG chains. As the result, the structure of SiAu-3D can be precisely and consistently controlled by tuning the size of SiNPs and AuPEG. In contrast, in the case of insufficient dosing of AuPEG, the formation of agglomeration or even aggregation can be observed, which is caused by cross-linking of SiNPs *via* AuPEG: unsaturated SiNPs has the tendency to share AuPEG with each other. The optimized conditions for the self-assembly of each nanostructure are listed in Table S1.†

Interestingly, the obtained SiAu-3D nanostructure shows no change in the UV-Vis absorption spectrum compared to pure AuPEG (Fig. S3†). The absence of a plasmonic coupling event indicates a large AuNP interparticle distance at the SiNP surface. The underlying reason is that AuNPs distribute evenly on the silica surface in a 3D fashion which meets our expectation from an isotropic colloidal self-assembly process. The uniform formation of the SiAu-3D structure is further confirmed by dynamic light scattering (DLS) measurements. A narrow single peak for SiAu-3D in THF with a hydrodynamic diameter slightly larger than both SiNPs and AuPEG is observed (Fig. 2B).

In addition, the selection of solvent is also a crucial parameter for this step. For instance, the self-assembly does not proceed well in water (Fig. S9†). This fact can be explained by the high degree of hydration of both SiNPs and AuPEG *via* hydrogen bonds in water, which hinder the formation of bonds between SiNPs and AuPEG. It can rationally be envisioned that the absence of hydrogen bonds between the PEG chain and solvent (*e.g.*, in THF) is critically important for an enthalpy-driven self-assembly process.

### Adsorption-based particle purification

**Fig. 3:**
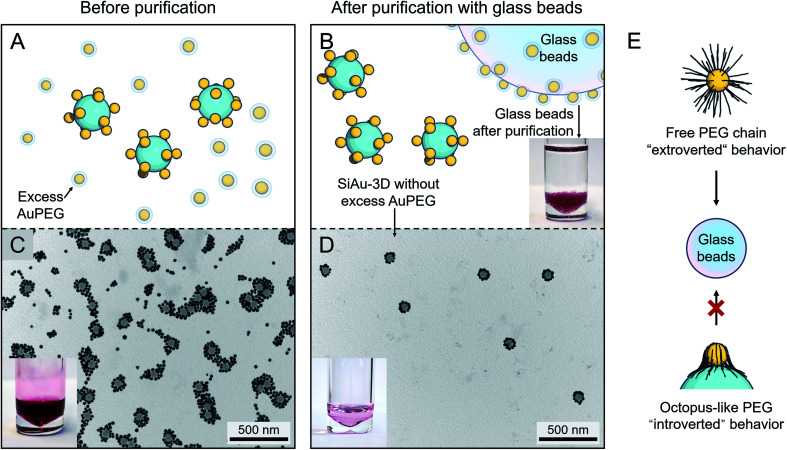
(A and B) Schematic illustration of the purification of SiAu-3D (removal of excess AuPEG) based on glass beads adsorption. The photograph in (B) shows the glass beads after purification steps placed in clean THF, no dissociation of AuPEG into THF can be observed. (C) TEM image and photograph of the unpurified mixture containing SiAu-3D and excess AuPEG. (D) TEM image and photograph of the SiAu-3D after purification. (C) and (D) are exemplary images corresponding to sample l. More TEM images verifying the purification results across all samples are shown in the ESI (Fig. S5 and S6†). (E) Schematic illustration of different PEG chain behavior on the free AuPEG (extrovert chain dynamic, strong adsorption onto glass surface) and on the SiAu-3D (“octopus”-like adsorption on SiNP surface, introverted behavior).

After the self-assembly process, excess AuPEG must be thoroughly removed to obtain the pure SiAu-3D for patterning. For this task, the commonly used centrifugation approach only shows insufficient separation performance, since the amount of excess AuPEG is considerably large. To tackle this problem, we developed a new method based on the adsorption of AuPEG upon glass beads (as illustrated in Fig. 3A and B). This idea originated from the observation that AuPEG has very strong adsorption on glass surface (Fig. S12†). By mixing and incubating the mixture of SiAu-3D and the excess AuPEG with the precalculated amount of glass beads, we can effectively remove all the residual AuPEG leaving the majority of SiAu-3D nanostructure in the colloid, as first evidenced by the transfer of red coloration (AuPEG) from the colloid to the glass beads after purification (photographs in Fig. 3B–D). TEM (transmission electron microscopy) images (Fig. 3C, D, S5 and S6†) clearly show the complete removal of AuPEG in all samples. DLS analysis also shows a narrow single peak for SiAu-3D (Fig. S4†) compared with a broad peak of the mixture of SiAu-3D and AuPEG before purification. Here, the successful isolation of SiAu-3D further indicates the much lower adsorption properties of SiAu-3D on the glass surface. The dramatically different adsorption behavior between SiAu-3D and AuPEG highly likely originates from the chain dynamics of the PEG brush. As illustrated in Fig. 3E, for AuPEG, the free PEG chains tend to adsorb onto glass surface (extroverted behavior) while the PEG chains on SiAu-3D are already attached to the silica surface extending themselves flatly (“octopus-like”), thus losing their affinity towards glass (introverted behavior).

Practically, this adsorption-based separation/cleaning technique offers a flexible and reliable method to remove AuPEG without any additional procedures. The handling of this process is extremely simple, since the AuPEG are so firmly attached to the surface of the glass beads that they cannot be redispersed back to THF (photograph in Fig. 3B). Furthermore, in the case of incomplete purification, adding several more glass beads can quickly clean up the residual AuPEG. The purified SiAu-3D colloid can be stocked over months without any structural change.

### 2D structural rearrangement

After purification, the SiAu-3D colloid is directly drop-cast on a carbon film for TEM analysis (Fig. 2C and S6†). Unlike the 3D nanostructure in the colloidal state, the AuNPs are now surrounding the SiNPs in a clear 2D circular pattern on the substrate (SiAu-2D). The AFM (atomic force microscopy) measurement (Fig. S7†) further shows that AuNPs are located around the base (meniscus region) of SiNPs. Such dramatic change of the internal structure indicates a substantial mobility of the AuPEG on the surface of silica NPs: the surface drifting of AuPEG on the SiNP surface needs to occur since the pathway from the top of a SiNP to the meniscus region is far longer than the contour length^36^ of the PEG chains (calculation in ESI, Fig. S8†). The hydrogen bonding between PEG and the SiNPs surface offers adequate adsorption to keep the AuPEG attached to the surface while at the same time providing sufficient mobility for moving around on the SiNPs surface allowing for the 3D-to-2D rearrangement. The AuPEG behaves just like an octopus with multiple arms latching on a core while simultaneously being able to drift/crawl along the surface. The driving force of this unique geometric evolution can be explained by surface energy minimization: the high attractive capillary force^37^ from the thin interface geometry is able to pull moveable PEG brushes into the meniscus region between the silica sphere and planar substrate during solvent evaporation. Since PEG are firmly anchored on the Au surface, AuNPs are also pulled towards the meniscus area resulting in the structural rearrangement.

**Fig. 4:**
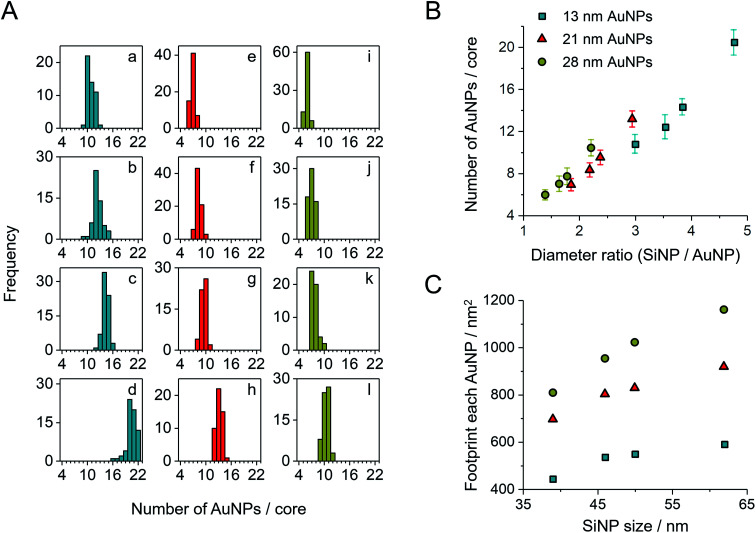
(A) Statistic evaluation of the number of AuNPs on each SiAu nanocluster. The sample labeling corresponds to Fig. 2C. (B) Relationship between the absolute number of AuNPs on each SiAu nanocluster and the diameter ratio between SiNP template and AuNPs. (C) Footprint (the surface area of SiNP divided by the number of AuPEG on each SiAu nanocluster) of AuPEG on SiNPs for each sample. The results of (B) and (C) are evaluated from the statistical data of (A).

This octopus-drift behavior of AuPEG is a comprehensive model to program the exact ring pattern of AuNPs. The control principle here is to exploit the effect of spatial saturation of AuPEG upon the surface of SiNPs in order to manipulate the number of AuNPs that matches the perimeter of the SiNPs. Fig. 2C shows a series of successful AuNPs-2D samples, which build up a library of ring nanopatterns of AuNPs. To the best of our knowledge, this is the first time that a precise control of the numbers of satellite nanoparticles on each core is achieved. The number of AuNPs on each SiNP template was statistically evaluated across all samples (Fig. 4A and Table S2†). The narrow distribution clearly originates from the spatial saturation of AuPEG on SiNPs. We can see that for the same size of AuNPs, increasing the size of the SiNP template will increase the number of attached AuPEG, since the total surface of silica is growing. The same logic leads to the explanation for the samples with the same SiNP size: the adsorbed number of AuPEG decreases by the increasing AuNP size, since the larger AuNP can carry more PEG chains.

We further systematically calculated the average occupied surface area of AuPEG on SiNP for each sample (*i.e.*, the total surface area of SiNPs divided by the number of attached AuPEG, for brevity, we call this value “footprint”). As result, we can see that the footprint increases significantly with the growing size of AuNP (Fig. 4C). In addition, the footprint of the same AuPEG slightly enlarges with increasing size of the template SiNPs, which possibly is caused by the decreased curvature of larger SiNPs.

Notably, the chain length of PEG also has a direct structural impact on the SiAu nanocluster, since the footprint of AuPEG on silica is directly dependent on the size of the attached PEG. If a shorter PEG chain is used (800 g mol^−1^), more AuPEG can be attached to the same silica core. As a result, the SiAu nanocluster cast to the surface will more retain its 3D characteristics, since the perimeter cannot accommodate all AuNPs (Fig. S10†). Consequently, only PEG with a molecular weight of 6000 g mol^−1^ was systematically applied in our work. The power of our approach also includes the fact that with only one single type of PEG brush, well-defined AuNPs rings with a wide size variation of SiNPs and AuNPs can be created. Fig. 4B clearly demonstrates the relatively uniform relation between the diameter ratio and the number of AuNPs in each SiAu-2D nanocluster. Remarkably, we can observe overlapped AuNPs or even 3D-like structures at a high diameter ratio (Fig. 2C, sample c and d, respectively). All these combined results show the effective geometrical impact of the PEG chain length and the size of each nanocomponent on the formation of SiAu-2D. By intuitively adjusting the parameters of these building blocks, the SiAu-2D gallery can quickly be expanded at will.

### Selective removal of the silica template

**Fig. 5:**
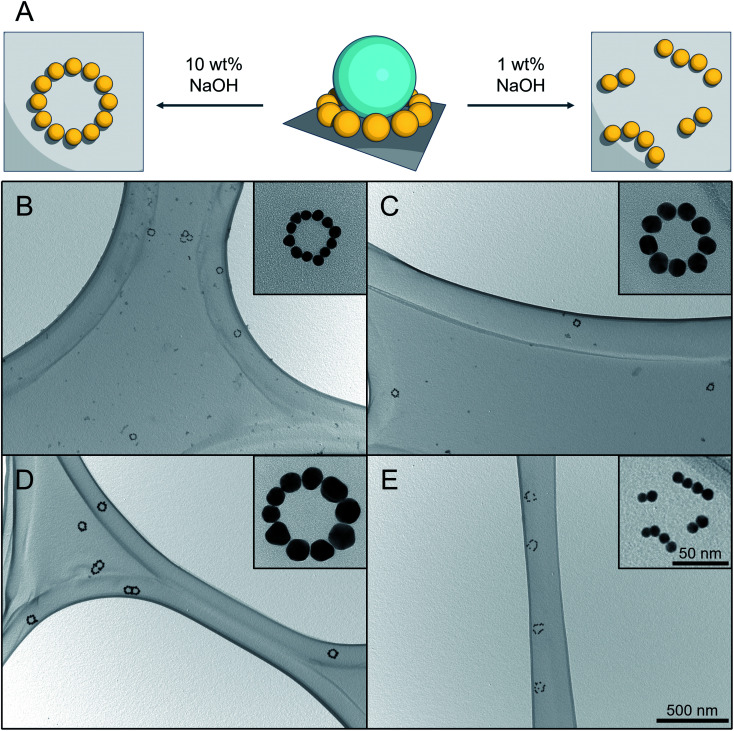
Result of silica etching under different conditions. (A) Schematic representation of silica etching with (left) immobilized AuNPs pattern by using 10 wt% aqueous NaOH solution, and (right) disordered pattern using 1 wt% aqueous NaOH solution. (B–D) TEM images of successful silica etching experiments using 10 wt% aqueous NaOH solution for different SiAu nanoclusters. The corresponding parental SiAu-2D nanostructures for (B), (C) and (D) are samples b, g, and l from Fig. 2C. (E) Result of silica etching of sample b using 1 wt% aqueous NaOH solution. The disordering of the ring patterns of AuNPs can clearly be observed for all of the clusters.

To simplify the structure and avoid any undesired optical disturbances caused by the large SiNP cores, we developed a straightforward protocol to selectively remove SiNPs, as illustrated in Fig. 5A.

Conventionally, there are several available options for silica etching, especially for nanoparticles, including HF,^38,39^ alkaline solution,^40,41^ or even hot water.^42^ The obvious concern here is the dissociation of AuPEG during the etching process, due to its excellent water dispersibility. Attempts made with 1 wt% NaOH aqueous solution,^40^ for instance, show a successful removal of the SiNPs, which, however, is unfortunately accompanied by an unwanted local disordering of the circular AuNPs patterns, as shown in Fig. 5E.

To address this issue, we established an innovative etching method by utilizing the ionic strength dependent LCST of PEG^43,44^ to immobilize AuNPs during the etching process. Practically, we utilize the fact that PEG has a significantly decreased solubility under high NaOH concentration.^45^ DLS analysis (Fig. S11†) shows that AuPEG is perfectly dispersible in water and diluted NaOH (1 wt%), while it is completely non-dispersible at high NaOH concentration (10 wt%). When incubating the substrate that is carrying SiAu-2D nanocluster at the surface with 10 wt% NaOH solution, the AuPEG is remaining fixed to the substrate while the silica etching proceeds effectively at room temperature. As a result, we obtain well-defined circular AuNPs nanopatterns across the whole substrate surface. The etching protocol works extremely well across the sample pool with its different ring diameters and AuNPs size, as demonstrated in Fig. 5B–D. Notably, at a raised temperature, the etching process can significantly be accelerated, while the ring of AuNPs still keeps its perfect geometry. A next step may be the further stabilization of the ring structures *via* cross-linking of the polymer, which may allow to detach them from the surface to the solution phase while remaining their complex structure. This, however, is beyond the scope of the present study.

## Conclusion

In summary, we demonstrated a new concept for colloidal self-assembly of nanoparticles by utilizing hydrogen bonds between polymer and silica surfaces. Polymer-covered Au nanoparticles are designed to “latch” to silica nanoparticle cores in a controlled fashion, forming core–satellite structures. The hydrogen bonds provide AuPEG a unique surface mobility on SiNP. As a result, the surface-attached AuPEG can rearrange into a 2D ring pattern surrounding the SiNPs template when cast to a substrate. We created a gallery of such SiAu-2D pattern with a variation of ring size, AuNPs size, and number of attached AuNPs. We further introduced a new strategy to purify the SiAu-3D nanostructures from excess AuPEG nanoparticles by applying glass beads. Finally, the SiNP templates were completely removed without any disordering of the perfect AuNPs ring pattern by etching with 10 wt% NaOH. This work shows a series of advantages by utilizing hydrogen bonds as a dynamic anchoring tool for creating nanoclusters with rearrangement ability. We believe that such concept will quickly expand its design options, thus extending the portfolio of innovative nanostructures.

## Experimental procedures

The experimental section is provided in the ESI.†

## Author contributions

The manuscript was written by contributions of all authors. All authors have given approval to the final version of the manuscript.

## Conflicts of interest

There are no conflicts to declare.

## Supplementary Material

NA-004-D2NA00204C-s001
