# Supplementary material for: Gold nanoparticle ring arrays from core–satellite nanostructures made to order by hydrogen bond interactions
Source: Nanoscale Adv. 2022 Apr 23;4(13):2787–93. doi: 10.1039/d2na00204c (PMC9417049; doi:10.1039/d2na00204c)
Supplement: NA-004-D2NA00204C-s001 [file NA-004-D2NA00204C-s001.pdf]

Electronic Supplementary Material (ESI) for Nanoscale Advances.
This journal is © The Royal Society of Chemistry 2022

Gold Nanoparticle Ring Arrays from Core–Satellite Nanostructures Made to Order by Hydrogen Bond Interactions

Supplementary information

Yingying Cai, Wentao Peng, Philipp Vana*

Institut für Physikalische Chemie, Georg-August-Universität Göttingen, Tammannstrasse 6, 37077 Göttingen, Germany.

*Corresponding Author: pvana@uni-goettingen.de

Experimental procedures

Chemicals. IGEPAL CO-520, tetraethyl orthosilicate (≥ 99%), trisodium citrate dihydrate (≥ 99%), NaOH (reagent grade), ethanol (99.8%), poly(ethylenglycol)methyletherthiol with molecular weight of 6000 and 800 g/mol, glass beads with diameter of 0.5 mm, and gold-coated silicon wafer were purchased from Sigma-Aldrich. Cyclohexane (≥ 99%), aqueous ammonia (~32%), and tetrahydrofuran were purchased from VWR. $HAuCl_4 \cdot 3H_2O$ (99.9%) was purchased from abcr. All chemicals were used as received. Ultrapure (type I) water (conductivity 18.2 MΩ · cm) was used for all experiments.

Synthesis of PEG capped AuNPs. The 13 nm AuNPs were synthesized using the citrate reduction method from literature.[1] A hot solution of sodium citrate (25 mL, 39 mM) was added to a boiling solution of $HAuCl_4$ (500 mL, 0.5 mM) under vigorous stirring. The mixture was refluxed for further 15 minutes and cooled to room temperature. The 13 nm AuNPs were used as seed for the synthesis of 21 and 28 nm AuNPs with a modified protocol from literature.[2] For the synthesis of 21 nm AuNPs, A mixture of 40 mL as-synthesized 13 nm AuNPs, 108 mL ultrapure water, and sodium citrate solution (1.13 mL, 60 mM) was kept stirring at 90 °C for 10 min before addition of aqueous $HAuCl_4$ solution (1 mL, 25 mM). The AuNPs were allowed to grow for 30 min at 90 °C before another addition of $HAuCl_4$ (1 ml, 25 mM). This cycle was repeated for another time to obtain ~21 nm AuNPs. For the synthesis of 28 nm AuNPs, 55 mL of as-synthesized 21 nm AuNPs were diluted with 53 mL water and additional sodium citrate solution (2.00 mL, 60 mM). The growth was proceeded again at 90 °C with three times addition of $HAuCl_4$ (1 ml, 25 mM) in 30 min interval. All above-mentioned AuNPs colloid can be stocked in polypropylene tubes under ambient condition for months without visible change.

To fabricate AuPEG, 120 mL colloid of citrate-capped AuNPs were mixed with an aqueous solution of poly(ethylenglycol)methyletherthiol (6 mL, 2 mg/mL for both chain length) under bath sonication. Four centrifugation (12000 rpm, 90 min) and redispersion cycles were applied to change the solvent from water to THF, remove excess PEG, and concentrate the colloid into the desired concentration. The AuPEG can be used as stock solution. **Fig. S1** shows the TEM images (capped with PEG) and corresponding size distribution of all types of AuNPs.

Synthesis of SiNPs. To fabricate SiNPs with high roundness, we applied a modified protocol based on reversed microemulsion technique[3]. SiNPs with different diameters are synthesized using the same microemulsion system, only the dosing of TEOS is variated. For 39 nm SiNPs, IGEPAL CO-520 (31.3 g) and cyclohexane (250 mL) were mixed under stirring for 10 min. Ammonia (32%, 2.3 mL) was then added dropwise to the solution and the mixture was stirred for another 30 min before the addition of TEOS (1.72 mL). The reaction proceeded for approximately 22 hours at room temperature under stirring. Ethanol (~50 mL) is added to the mixture to quench the reaction. The SiNPs were purified by three centrifugation cycles in ethanol (5000 rpm, 30 min) and finally dispersed and stocked in THF. For 46 nm, 50 nm and 62 nm SiNPs, additional TEOS (0.9 mL, 2 × 0.7 mL, 6 × 0.7 mL, respectively) was added to the mixture after each reaction step. The reaction time between each TEOS addition was 24 hours. The quenching and purification were performed using the same method and condition. **Fig. S2** shows the TEM images and the corresponding size distribution of all types of SiNPs.

Colloidal self-assembly of SiAu-3D. In general, the experimental procedures of colloidal self-assembly of SiAu-3D with different AuPEG and SiNPs are similar. The only difference is the dosing of AuPEG and SiNPs. Here an example of 13 nm AuNPs and 39 nm SiNPs is given to demonstrate the procedure. In a typical run, 27 µL of AuPEG sol (containing approximately 3 mgAu/mL in THF) was added into a colloid

of 39 nm SiNPs (120 µL, 0.1 mg/mL in THF) in a 1.5 mL glass vial under bath sonication. The mixture was allowed to be further sonicated for additional 30 minutes and then placed on an orbital shaker (150 rpm) overnight to ensure a saturation of AuPEG on the SiNPs surface. The detailed feed ratios for different SiAu-3D nanoclusters prepared in this work are summarized in **Tab. S1**.

Purification of SiAu-3D by glass beads. Based on our experience, the purification performance is highly dependent on the amount ratio between the glass beads and excess AuPEG rather than the concentration of the colloid. In this way, we can estimate the amount of glass beads for each type of AuPEG with different AuNPs sizes. We further found that the Ø 0.5 mm glass beads have the most balanced performance and handling property thus suitable for all types of AuPEG used in this work. In general, 12, 4, and 5 mg glass beads are sufficient to remove 1 µg of 13, 21, 28 nm-sized AuNPs (the weight of PEG shell is ignored here). In a typical run, glass beads were added in two steps to the unpurified nanocluster colloid. Extra THF was added to keep all glass beads below the liquid level. After each addition step of the glass beads, the sample was placed on an orbital shaker (175 rpm) for 20 minutes. The purified colloid was separated from the glass beads to a clean glass vial by a micropipette. TEM characterization was employed to control the purification result. If any free AuPEG remains, an additional purification step can be executed by using a decreased amount of glass beads to achieve the ideal purification result.

Notably, for the sample with shorter PEG chains (800 g/mol, 13 nm AuNPs), only a 25% dosing of glass beads is required to completely remove all AuPEG.

Formation of SiAu-2D on substrate. To bring SiAu nanocluster from colloid onto a substrate, we simply drop-cast 10 µL of SiAu-3D colloid onto a carbon film-covered TEM grid. The solvent was allowed to evaporate under ambient conditions. For silica removal application, lacey carbon film was used instead of the common carbon film for better liquid penetration which yields more consistent etching results. For AFM characterization, 2 µL of SiAu-3D colloid was drop-cast on a piece of gold-covered silicon wafer. The substrates were covered with a glass vial and left undisturbed under ambient conditions overnight to ensure a complete evaporation of the solvent.

Selective removal of SiNP template. TEM grid (lacey carbon film) carrying SiAu-2D at the surface was incubated in a 10 wt% aqueous NaOH solution overnight. After incubation, the TEM grid was gently dipped in water and ethanol successively to remove any remaining NaOH. The TEM grid was then left undisturbed for solvent evaporation before TEM characterization.

Characterizations. TEM imaging was performed on a Philips CM 12 electron microscope. The TEM was operated at an acceleration voltage of 120 kV and an emission current of 3–4 µA. The DLS was performed on a Malvern Zetasizer Nano S system operating at 633 nm at 25 °C. Samples were measured after a 120 s equilibration period within the analyzer without previous filtration. UV-Vis absorption spectroscopy was performed with a Jasco V770 scan photo spectrometer in solution against pure solvent (baseline subtraction method). A halogen lamp was used as the light source. AFM measurement was carried out on a Bruker Multimode 8 equipped with a Scanasyst-Air-HR cantilever.

Figures and Tables

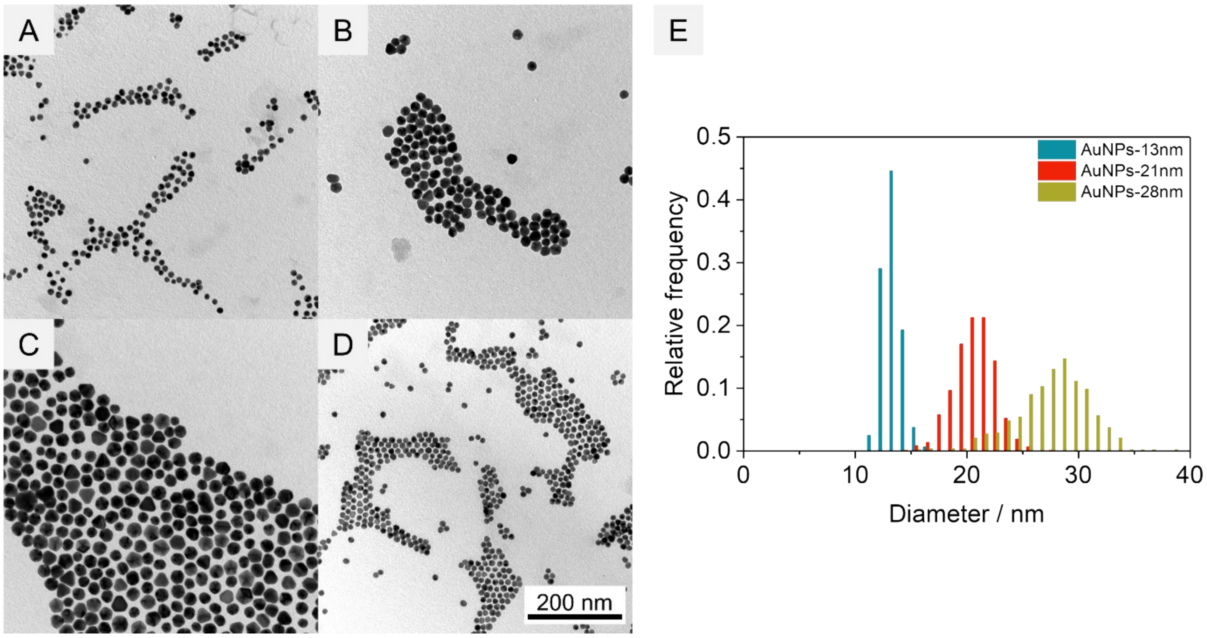

Fig. S1 **(A–C)** TEM images of 6000 g/mol PEG capped AuNPs with diameters of (A) 13 ± 1 nm, (B) 21 ± 2 nm, and (C) 28 ± 3 nm, respectively. **(D)** 13 nm AuNPs capped with 800 g/mol PEG. **(E)** Size distribution of 3 types of AuNPs. Each sample is evaluated by over 450 particles. The errors represent the standard deviation.

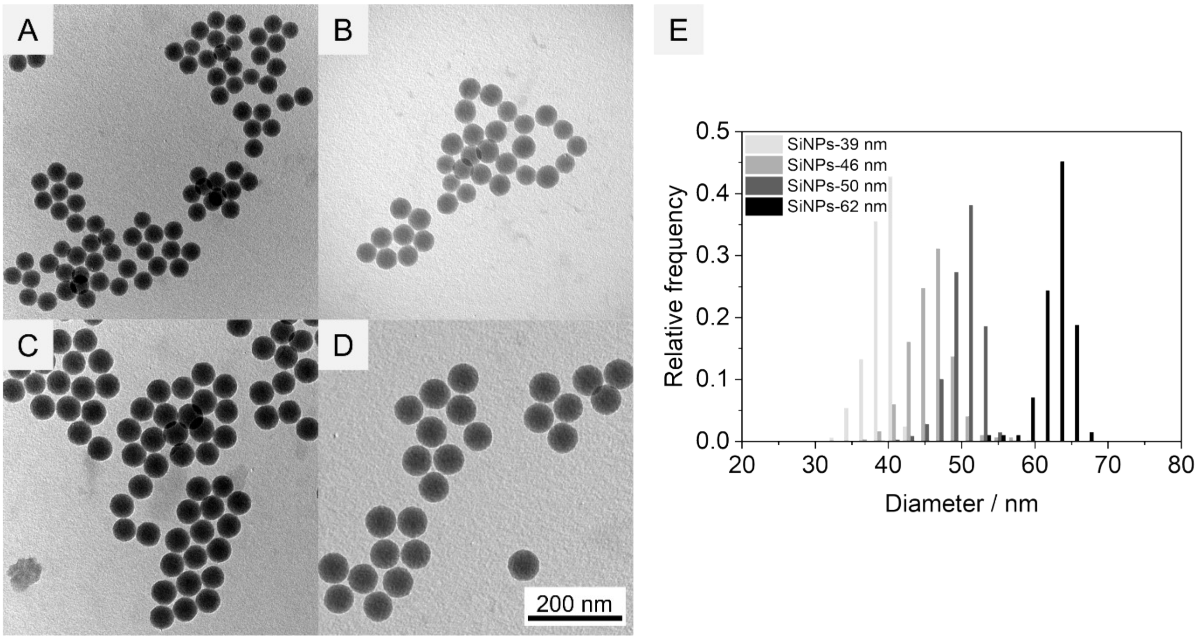

Fig. S2 TEM images of as-synthesized SiNPs with diameters of **(A)** 39 ± 2 nm, **(B)** 46 ± 3 nm, **(C)** 50 ± 2 nm, and **(D)** 62 ± 2 nm, respectively. **(E)** The corresponding size distributions of SiNPs. Each sample is evaluated by over 200 particles. The errors represent the standard deviation.

Tab. S1 Detailed experimental parameters for self-assembly process of SiAu nanoclusters with different SiNPs and AuPEGs. For the calculation, the density of AuNPs is 19.3 g/cm³, the density of SiNP is 2.2 g/cm³. The diameter of each NP is determined from TEM statistically (**Fig. S1–S2**). The concentration of AuPEG is calculated under the assumption that no material loss occurs during the centrifugation process.

AuNPs diameter (nm)	SiNPs diameter (nm)	PEG shell molecular weight (g/mol)	Approx. AuPEG concentration (mgAu/mL)	AuPEG volume (µL)	Number of AuNPs	SiNPs concentration (mg/mL)	SiNPs volume (µL)	SiNPs number	Number ratio (AuNPs/SiNPs)
13	39	6000	3	27	3.6×10^{12}	0.1	120	1.8×10^{11}	21
	46		3	27	3.6×10^{12}	0.1	120	1.1×10^{11}	34
	50		3	45	6.1×10^{12}	0.2	120	1.7×10^{11}	36
	62		3	70	9.5×10^{12}	0.4	120	1.7×10^{11}	54
21	39		6.7	45	3.2×10^{12}	0.1	120	1.8×10^{11}	18
	46		6.7	55	3.9×10^{12}	0.1	120	1.1×10^{11}	37
	50		6.8	75	5.4×10^{12}	0.2	120	1.7×10^{11}	33
	62		6.8	105	7.6×10^{12}	0.4	120	1.7×10^{11}	44
28	39		16.6	38	2.8×10^{12}	0.1	120	1.8×10^{11}	16
	46		16.6	20	1.5×10^{12}	0.1	120	1.1×10^{11}	14
	50		16.6	45	3.4×10^{12}	0.12	200	1.7×10^{11}	20
	62		16.6	60	4.5×10^{12}	0.24	200	1.7×10^{11}	26
13	39	800	2	180	1.6×10^{13}	0.1	120	1.8×10^{11}	92

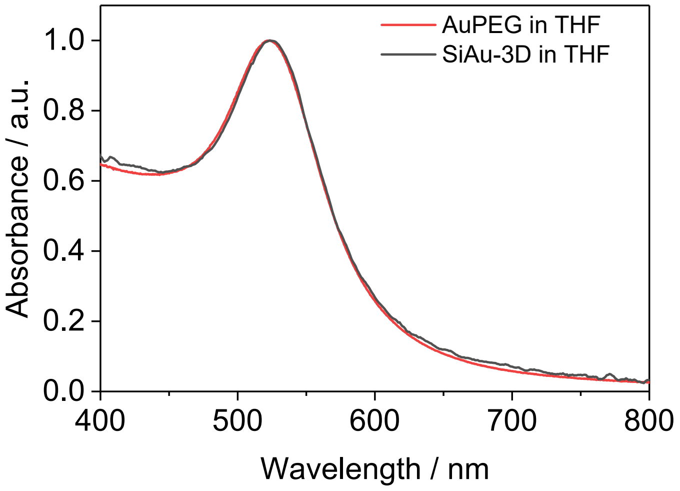

Fig. S3 UV-Vis spectra of AuPEG in THF (red curve) and SiAu-3D (after purification step) with 13 nm AuNPs and 46 nm SiNPs (black curve). The spectra are normalized at peak maximum (523 nm).

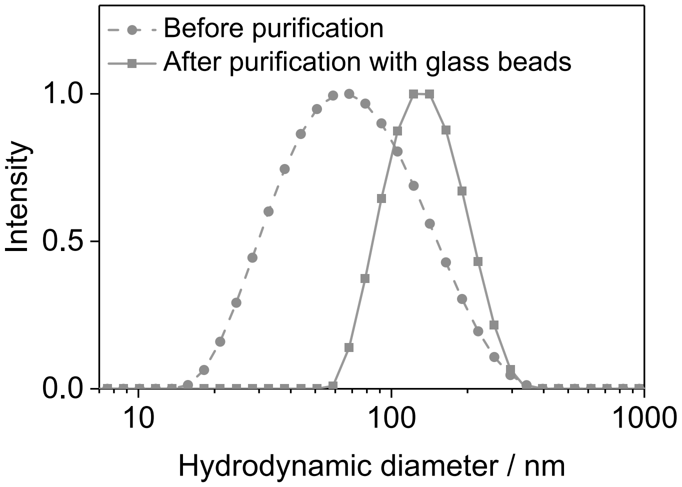

Fig. S4 DLS results of SiAu-3D (using 46 nm SiNPs and 13 nm AuNPs) in colloid before and after purification procedures.

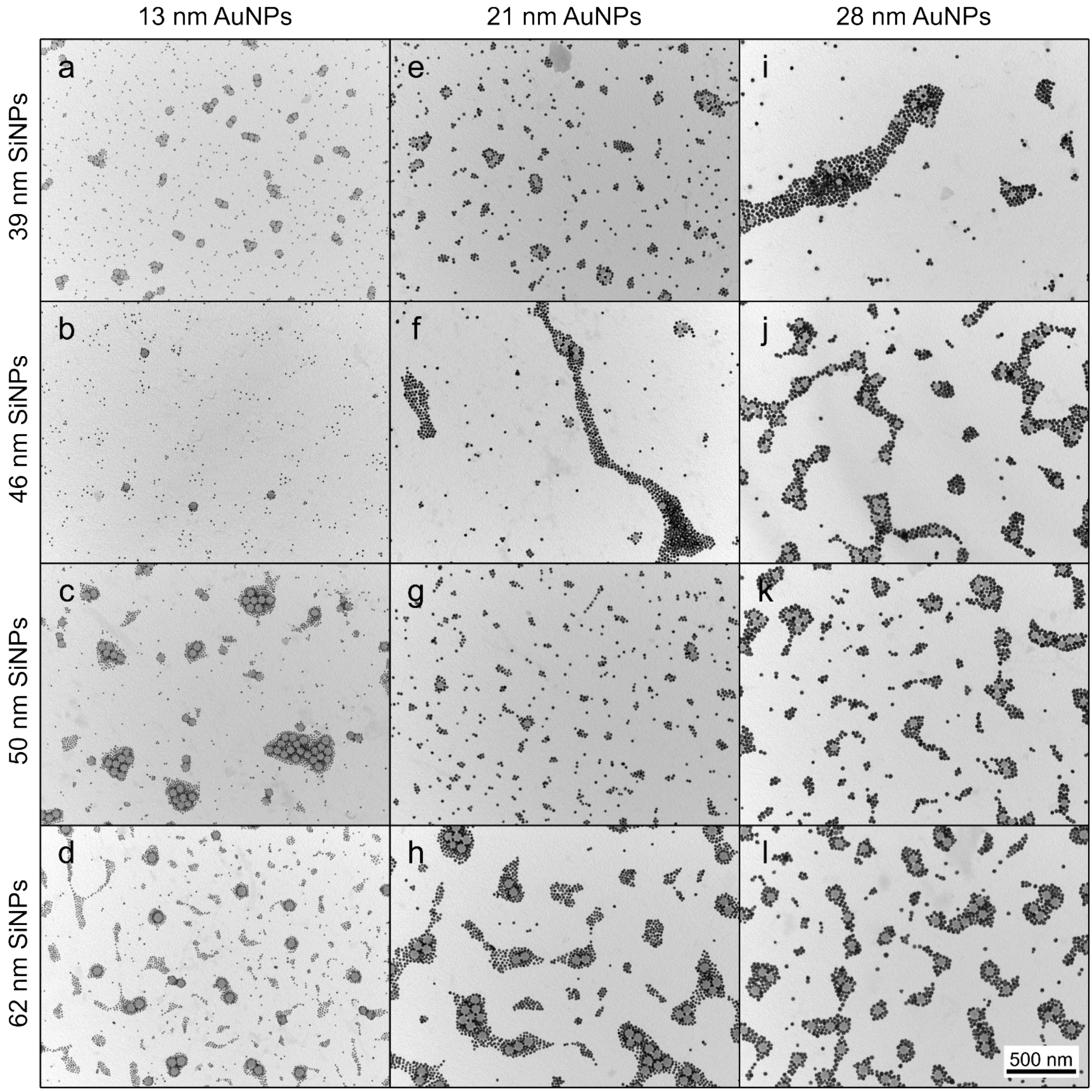

Fig. S5 TEM images of SiAu-2D nanoclusters from different sizes AuNPs and SiNPs containing excess AuPEG (before purification). The sample labeling is corresponsive to **Fig. 2C** in the main text.

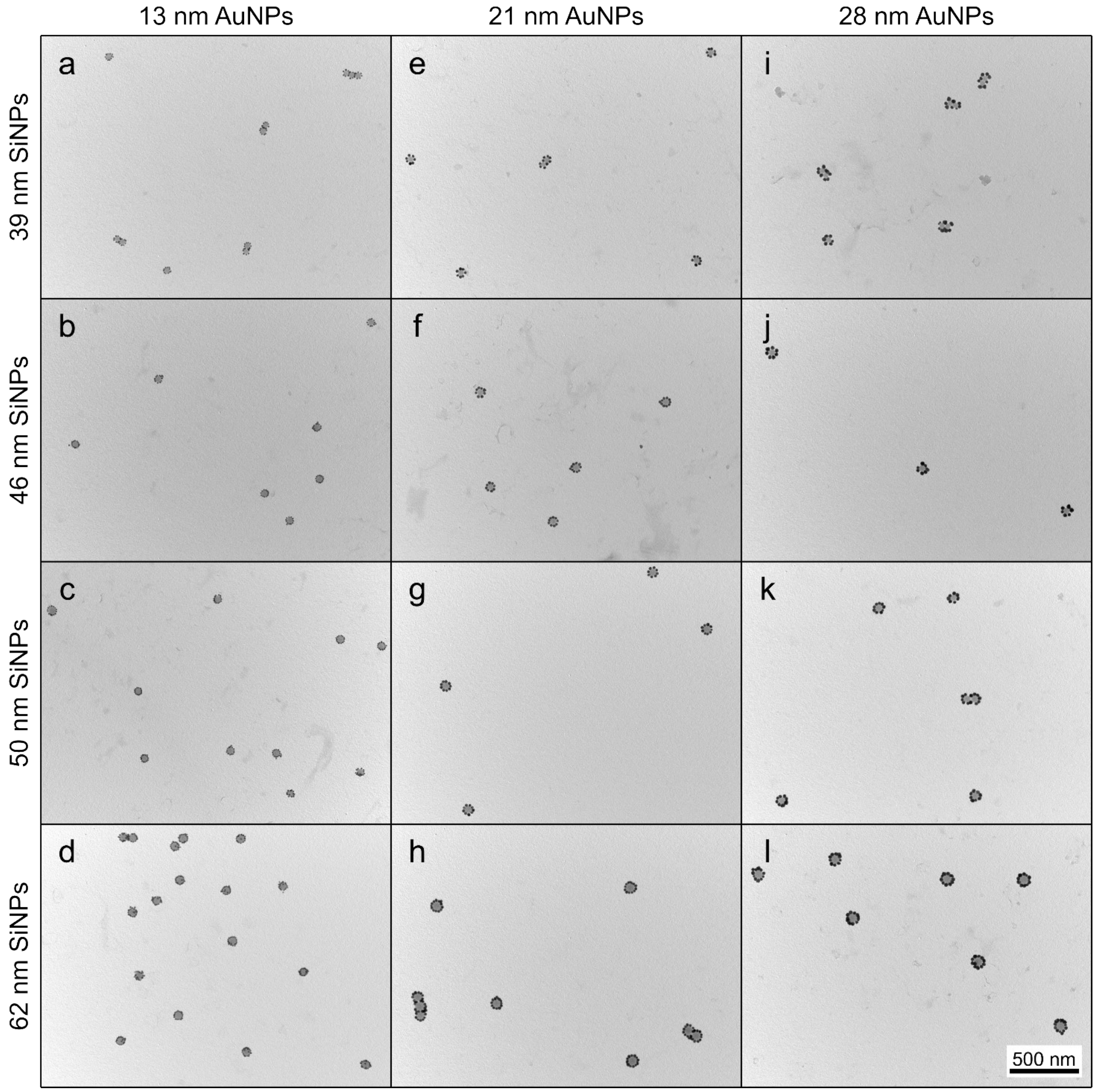

Fig. S6 TEM images of SiAu-2D nanoclusters (purified with glass beads) from different sizes AuNPs and SiNPs. The sample labeling is corresponsive to **Fig. 2C** in the main text.

Tab. S2 Statistics of AuNPs number in each single nanocluster corresponding to sample a–l. These values are obtained by evaluating over 48 clusters for each sample in TEM images.

	**13 nm AuNPs**	**21 nm AuNPs**	**28 nm AuNPs**
**39 nm SiNPs**	10.8 ± 0.9	6.9 ± 0.6	5.9 ± 0.5
**46 nm SiNPs**	12 ± 1	8.3 ± 0.7	7.0 ± 0.7
**50 nm SiNPs**	14.3 ± 0.8	9.5 ± 0.7	7.7 ± 0.8
**62 nm SiNPs**	21 ± 1	13.1 ± 0.8	10.4 ± 0.8

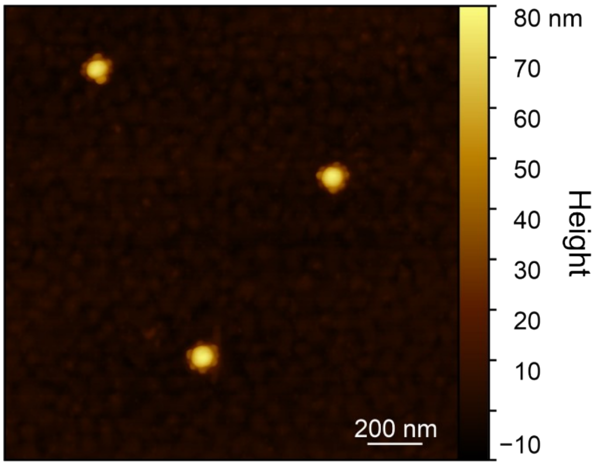

Fig. S7 AFM image of SiAu-2D nanocluster (with 62 nm SiNPs and 28 nm AuNPs) measured on a gold-coated silicon surface.

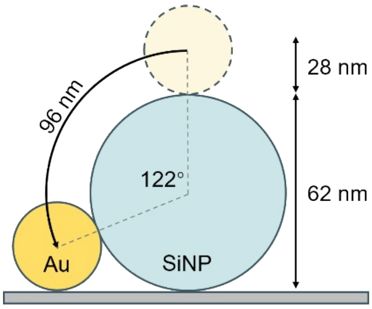

Fig. S8 Schematical illustration of exemplary travel distance (center of mass) of a 28 nm AuNP (yellow sphere) from the top of a 62 nm SiNP (cyan sphere) to the meniscus area of the silica-substrate interface. The travel distance is calculated by geometry: $(31\,nm\ +\ 14\,nm)\cdot(2\pi\cdot 122°/360°)\approx 96\,nm$. This value is much longer than the contour length of a 6000 g/mol PEG chain (38 nm, calculated from 0.28 nm for the contour length of each monomer unit[4].

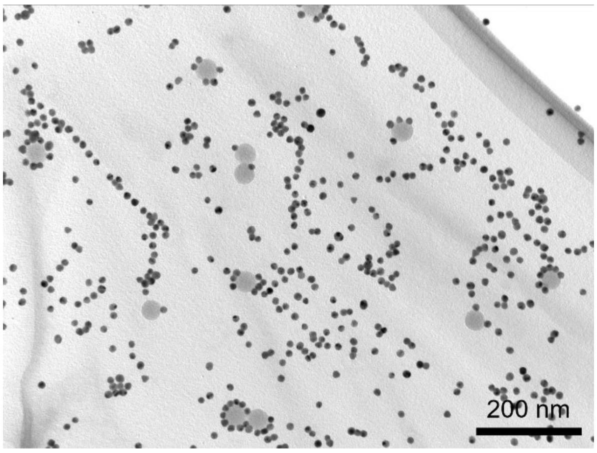

Fig. S9 AuPEG (with 13 nm AuNPs) and SiNPs (39 nm) assembled in water with the same dosing and handling conditions used for self-assembly in THF. Inefficient self-assembly result in water was observed.

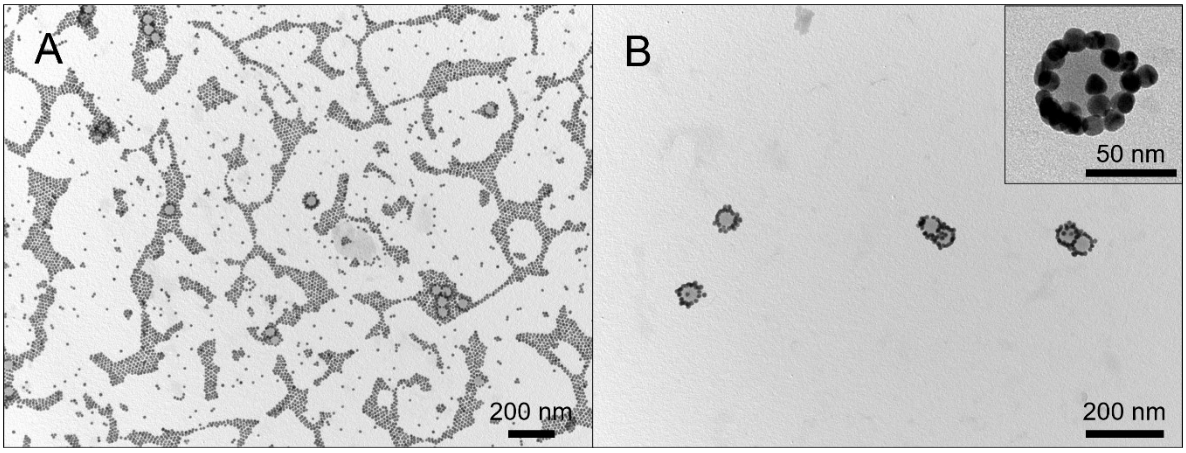

Fig. S10 TEM images of SiAu nanoclusters (13 nm AuNPs, 39 nm SiNPs) by using short PEG (800 g/mol) **(A)** before purification and **(B)** after purification procedures.

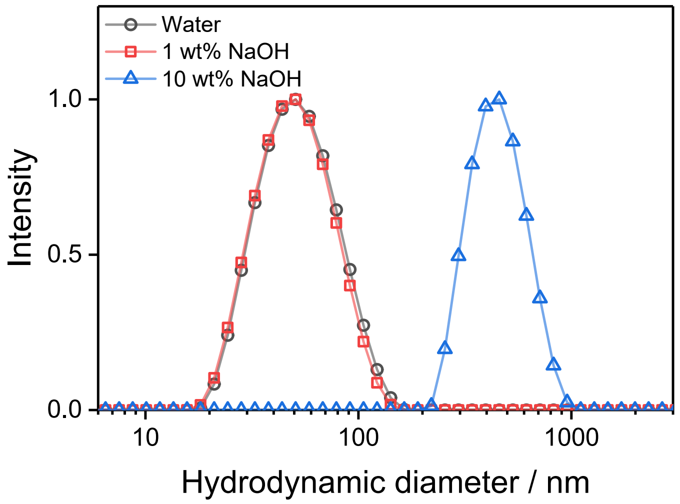

Fig. S11 DLS results (intensity distribution) for 0.01 mg/mL AuPEG (with 13 nm AuNPs) in water, in 1 wt%, and 10 wt% aqueous NaOH solution at 25 °C. The dramatically increased hydrodynamic diameter of AuPEG in 10 wt% NaOH solution indicates the undispersed behavior of AuPEG.

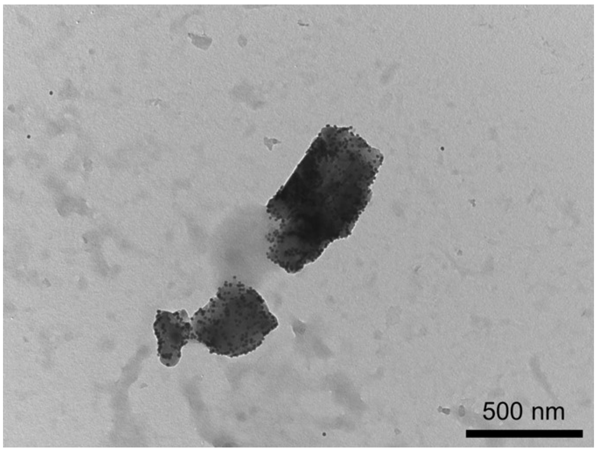

Fig. S12 TEM image of AuPEG (dispersed in THF, with 13 nm AuNPs) adsorbed on the glass powder surface. Each glass surface can adsorb a great number of AuPEG which validates the high efficiency of glass beads-based purification approach.

References

1 W. Peng, C. Rossner, V. Roddatis and P. Vana, *ACS Macro Lett.*, 2016, **5**, 1227–1231.

2 N. G. Bastús, F. Merkoçi, J. Piella and V. Puntes, *Chem. Mater.*, 2014, **26**, 2836–2846.

3 Y. Cai, W. Peng, S. Demeshko, J. Tian and P. Vana, *Macromol. Rapid Commun.*, 2018, **39**, 1800226.

4 F. Oesterhelt, M. Rief and H. E. Gaub, *New J. Phys.*, 1999, **1**, 6.
